# Pediatric Floating Elbow Caused by a Novel Mechanism: A Case Report

**DOI:** 10.7759/cureus.29124

**Published:** 2022-09-13

**Authors:** Mustafa Y Albattat, Hisham Alhathloul, Mohammed Almohammed Saleh, Fatimah Althabit

**Affiliations:** 1 Orthopedic Surgery, King Fahad Specialist Hospital, Dammam, SAU; 2 Orthopedic Surgery, King Fahad General Hospital, Hofuf, SAU; 3 Orthopaedic Surgery, King Fahad Hospital, Al-Ahsa, SAU; 4 Orthopaedic Surgery, King Fahad General Hospital, Al-Ahsa, SAU; 5 Medicine, King Faisal University, Al-Ahsa, SAU

**Keywords:** impending, acute compartment, compartment, supracondylar humeral fracture, humeral, pediatric fractures, floating elbow

## Abstract

A pediatric floating elbow is a rare condition in which there is a humeral supracondylar fracture with an ipsilateral fracture of one or both bones of the forearm. We report a case caused by an unusual mechanism of a semi-automatic washing machine. This injury, on its own, involves the risk of compartment syndrome, however, this particular child came late. We present our approach to this challenging injury along with the controversial management in the literature.

## Introduction

A pediatric floating elbow is a humeral supracondylar fracture with an ipsilateral fracture of one or both bones of the forearm [1]. This type of injury is complex trauma, which is believed to be associated with serious neurological and vascular complications. On top of these complications is acute compartment syndrome; this is thought to be a sequela of conservative management [2,3]. The floating elbow is an unusual trauma, especially in children, accounting for 3-13% of supracondylar fractures. The most common mechanism of injury was a fall from a height [4-6]. Road traffic accidents were the second most common and consist of 4-6 % of reported cases [7,8]. We present a case of a child who presented with an atypical mechanism of the floating elbow, caused by a washing machine. The patient underwent close-reduction internal fixation with titanium elastic nails (TENs). After six months of follow-up, bone healing was seen in the radiographs and the elastic nails were removed.

## Case presentation

**Figure 1:**
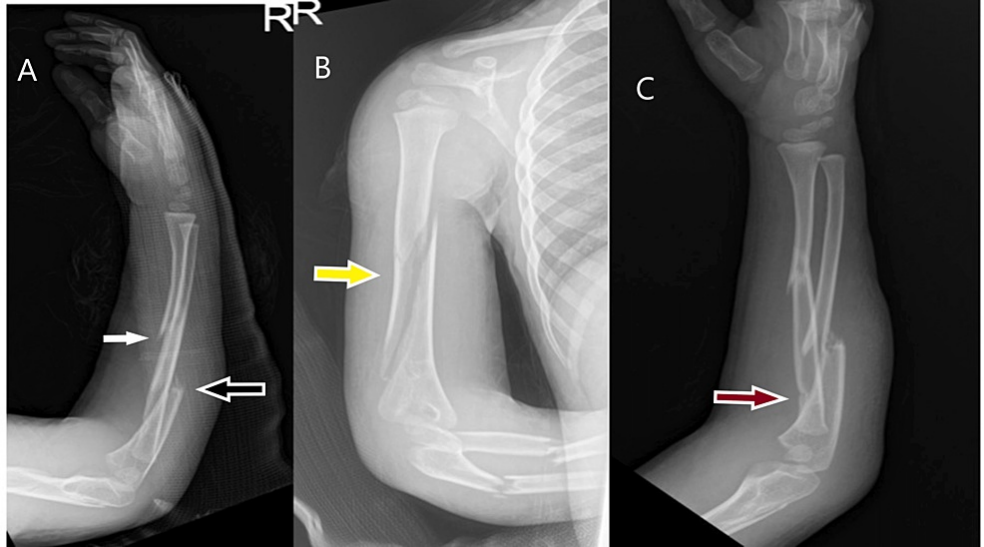
Initial radiographs (A) White arrow pointing to the radius fracture and black to the ulnar fracture, (B) Yellow arrow pointing to the long oblique humeral fracture with the lateral butterfly fragment, (C) Red arrow showing another fracture line in the proximal radius

**Figure 2:**
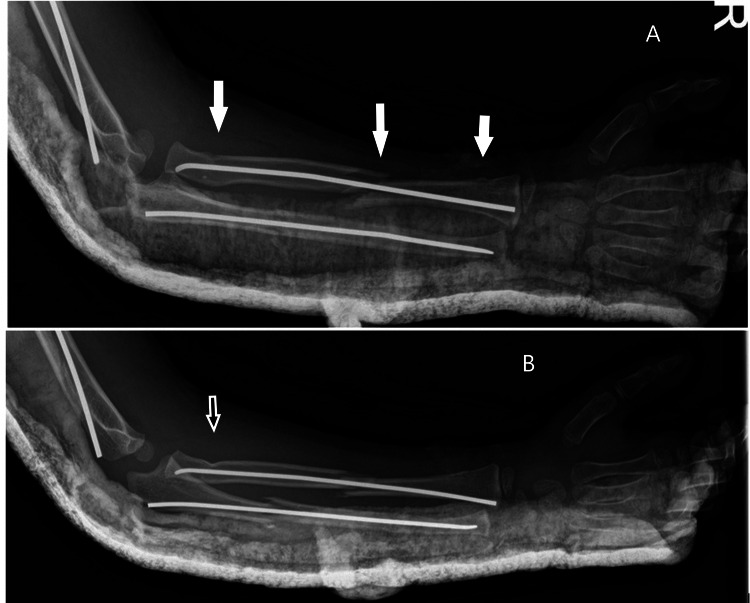
Postoperative radiographs of the forearm (A) Showing elastic nail fixation for forearm fractures with a temporary back slab, multiple fracture sites are pointed to by the white arrows; (B) showing the proximal radial fracture clearly in both cortices

**Figure 3:**
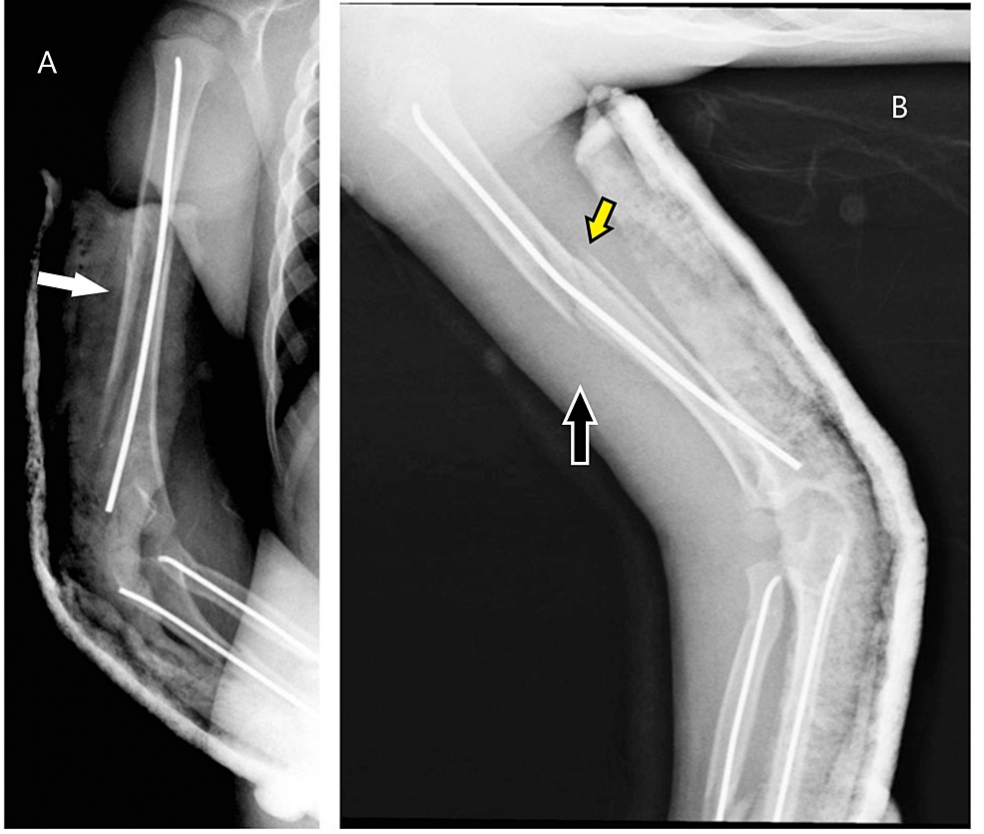
Postoperative radiographs (A) Titanium elastic nail fixation for humeral fractures with a temporary back slab without jeopardizing the butterfly fragment, white arrow; (B) Lateral view, the black arrow pointing to the nail bowing over the fracture site with good alignment and the yellow arrow pointing to the alignment of the long oblique fracture

**Figure 4:**
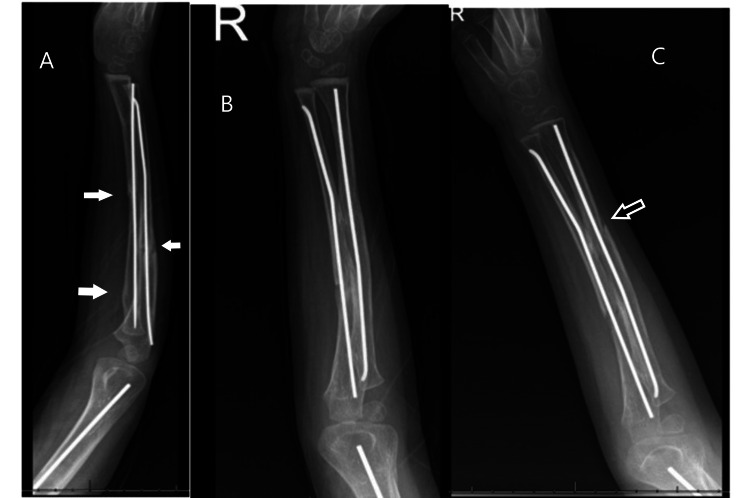
Follow-up radiographs of the forearm Good fracture healing in the forearm (A) Lateral view: white arrows pointing to the healed fracture; (B) Anterior-posterior view; (C) Oblique view: the black arrow showing a bridging callus for the radius fracture

**Figure 5:**
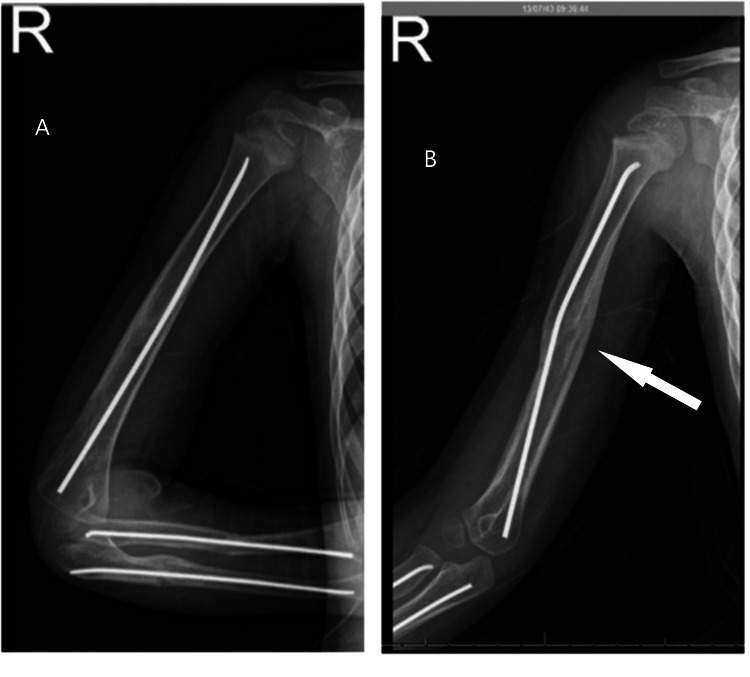
Follow-up radiographs for humeral fractures (A) Good fracture healing in the anterior-posterior view; (B) Different view with the white arrow pointing to the bridging callus

The patient was a five-year-old boy who presented to the emergency department with right arm and forearm pain. The pain started 12 hours prior to the presentation after putting his upper limb in a working semi-automatic washing machine. The pain was associated with an inability to move his right upper limb and marked swelling. On examination, his temperature was 36.5 Celsius, heart rate was 120 beats per minute, and respiratory rate was 27 times per minute. The blood pressure was 100/77 millimeters of mercury. Oxygen saturation was 100 % in room air. He was in significant pain and distress but conscious and alert. The affected limb was examined and evaluated. It was severely swollen, with scattered ecchymosis over the arm and forearm. The right upper limb was tender all over and felt tense on palpation. However, the neurovascular examination was normal. Realignment was done under conscious sedation and a back slab cast was applied. We did bedside portable radiographs to evaluate the injuries (Figure 1). The patient was taken directly to the operating room because we suspected impending compartment syndrome. We examined him under anesthesia. Acute compartment syndrome was excluded. We fixed both the forearm and humeral fractures. We started with the forearm fracture because it was a segmental fracture in the radius (Figure 2). Both forearm fractures were fixed with TENs. Then, the humeral fracture was reduced and fixed by a single retrograde TEN (Figure 3). The reduction was confirmed by X-rays. The patient was kept for 48 hours of observation with strict limb elevation and ice packing. We checked his compartment clinically every four hours. We discharged him and followed up with him every week for the first three weeks. After six months, when the fracture union was ensured completely, we booked him for the removal of the implants (Figures 4, 5). The functional outcomes were excellent, and no residual neurological or vascular complications were found.

## Discussion

The pediatric floating elbow fractures vary by the site of forearm fracture - the proximal, middle, and distal forearm. Distal forearm association was more prevalent with subdivision into metaphyseal and physeal distal radius fractures. The location of the fracture in the proximal forearm suggested more traumatic force [9,10]. Moreover, it could be an open or closed injury. The prevalence of open injuries varies from 13-22% and are more in humeral rather than forearm fractures [7,8,11,12]. In our case, we report a peculiar rotational injury by a semi-automatic washing machine. As far as we are aware, no similar mechanism was reported in the literature. Pediatric floating elbow injuries are believed to evolve compartment syndrome, which was estimated to be from 7-33% [3,13]. In our case, the patient came with an impending compartment due to a delay of more than 12 hours, which also could elevate the compartmental pressure [14]. Contrary to what was believed about the risk of compartment syndrome, some authors propose that it could be an overestimation and it was likely secondary to other factors rather than being from the pattern of the injury itself [12,14,15]. Hence, simultaneous ipsilateral forearm fractures and supracondylar fractures do not increase the latter's own risk of acute compartment syndrome [16]. Furthermore, the lack of significant reported cases of missed compartment syndrome in pediatric floating elbow injuries reinforces this assumption [17]. Although assessment of compartment syndrome is clinical judgment, It seems to be challenging in the pediatric population [18]. This problem was encountered in this case, so we preferred to take the patient to the operating room for proper examination under general anesthesia, reduction, and fixation.

There was no consensus on the treatment of floating elbow in the literature. So, the management varies from conservative by closed reduction and cast to operative or combined [19,20]. The role of conservative management is still a valid option in forearm fractures but the trend for humeral fractures was more with operative management [8,21,22]. However, displacement after immobilization was not uncommon, thus there was a risk of compartment syndrome especially if a circular cast was used [9,22]. The rate of displacement was estimated between 12% and 21%. However, the age, time of the reduction, and severity of initial displacement did not reproduce the displacement. Moreover, it usually occurs in the first or second week after the closed reduction [20,23]. Operative management has started to be the trend of treatment of choice for the majority of surgeons in the last two decades due to many factors [24-26]. Fixation of the humerus fracture provided better neurovascular protection and prevented cubitus varus [8,27], even though there was no difference in long-term follow-up in cubitus varus development with conservative management [28]. In our case, we started with forearm closed reduction and fixation because it was segmental, which was also rare [29]. We preclude conservative treatment for the forearm with merely circumferential immobilization to prevent increasing compartmental pressure. The priority of reduction and fixation based on whether forearm or supracondylar is still debatable [6,11]. Hence, we started with forearm fixation with TENs. Then, we reduced the humeral fracture with TENs as well because fracture geometry is different. It was a long spiral with an extension to the middle of the diaphysis. There was a big butterfly fragment laterally, which hindered the k-wires fixation. We used TENs in retrograde fashion as an internal splint to restore the alignment. The patient was kept for 48 hours under close monitoring with elevation and ice packing. We believed this method was functionally ideal. It prevented elbow stiffness as the patient could perform some sort of elbow movement. Moreover, it avoided injury of the ulnar nerve compared to percutaneous pinning [30-32]. Our report was limited, as it was a single case report and no similar rotatory mechanism of injury. The presence of a segmental forearm fracture with an ipsilateral humeral fracture made our report unique.

## Conclusions

In conclusion, soft tissue manipulation and treatment were crucial in this kind of injury, as the compartmental pressure was inherently elevated. Close follow-up was important in the first three weeks because the displacement was probable. The outcomes were good to excellent but complications could happen.

## References

[1] El Ibrahimi A, Shimi M, Daoudi A, Elmrini A (2012). Floating elbow: retrospective study and review of literature [Article in French]. Chir Main.

[2] Stanitski CL, Micheli LJ (1980). Simultaneous ipsilateral fractures of the arm and forearm in children. Clin Orthop Relat Res.

[3] Blakemore LC, Cooperman DR, Thompson GH, Wathey C, Ballock RT (2000). Compartment syndrome in ipsilateral humerus and forearm fractures in children. Clin Orthop Relat Res.

[4] Tabak AY, Celebi L, Muratli HH, Yağmurlu MF, Aktekin CN, Biçimoglu A (2003). Closed reduction and percutaneous fixation of supracondylar fracture of the humerus and ipsilateral fracture of the forearm in children. J Bone Joint Surg Br.

[5] Templeton PA, Graham HK (1995). The 'floating elbow' in children. Simultaneous supracondylar fractures of the humerus and of the forearm in the same upper limb. J Bone Joint Surg Br.

[6] Dhoju D, Shrestha D, Parajuli N, Dhakal G, Shrestha R (2011). Ipsilateral supracondylar fracture and forearm bone injury in children: a retrospective review of thirty one cases. Kathmandu Univ Med J (KUMJ).

[7] Mishra PK, Khare A, Gaur S, Gohiya A (2019). Paediatric floating elbow-a prospective study. J Clin Diagn Res.

[8] Harrington P, Sharif I, Fogarty EE, Dowling FE, Moore DP (2000). Management of the floating elbow injury in children. Simultaneous ipsilateral fractures of the elbow and forearm. Arch Orthop Trauma Surg.

[9] Roposch A, Reis M, Molina M, Davids J, Stanley E, Wilkins K, Chambers HG (2001). Supracondylar fractures of the humerus associated with ipsilateral forearm fractures in children: a report of forty-seven cases. J Pediatr Orthop.

[10] Blumberg TJ, Bremjit P, Bompadre V, Steinman S (2018). Forearm fixation is not necessary in the treatment of pediatric floating elbow. J Pediatr Orthop.

[11] Joshi RR, Dwivedi R, Byanjankar S, Shrestha R (2016). Simultaneous ipsilateral pediatric fractures of the elbow and forearm attending a tertiary care hospital. J Lumbini Med Coll.

[12] Baghdadi S (2020). Pediatric floating elbow injuries are not as problematic as they were once thought to be: a systematic review. J Pediatr Orthop.

[13] Williamson DM, Cole WG (1992). Treatment of ipsilateral supracondylar and forearm fractures in children. Injury.

[14] Battaglia TC, Armstrong DG, Schwend RM (2002). Factors affecting forearm compartment pressures in children with supracondylar fractures of the humerus. J Pediatr Orthop.

[15] Suresh S (2007). Management of "floating elbow" in children. Indian J Orthop.

[16] Muchow RD, Riccio AI, Garg S, Ho CA, Wimberly RL (2015). Neurological and vascular injury associated with supracondylar humerus fractures and ipsilateral forearm fractures in children. J Pediatr Orthop.

[17] Robertson AK, Snow E, Browne TS, Brownell S, Inneh I, Hill JF (2018). Who gets compartment syndrome?: a retrospective analysis of the national and local incidence of compartment syndrome in patients with supracondylar humerus fractures. J Pediatr Orthop.

[18] Bae DS, Kadiyala RK, Waters PM (2001). Acute compartment syndrome in children: contemporary diagnosis, treatment, and outcome. J Pediatr Orthop.

[19] Papavasiliou V, Nenopoulos S (1986). Ipsilateral injuries of the elbow and forearm in children. J Pediatr Orthop.

[20] Biyani A, Gupta SP, Sharma JC (19891). Ipsilateral supracondylar fracture of humerus and forearm bones in children. Injury.

[21] Yokoyama K, Itoman M, Kobayashi A, Shindo M, Futami T (1998). Functional outcomes of "floating elbow" injuries in adult patients. J Orthop Trauma.

[22] Illingworth KD, Meisel E, Skaggs DL (2019). The pediatric floating elbow. Oper Tech Orthop.

[23] Voto SJ, Weiner DS, Leighley B (1990). Redisplacement after closed reduction of forearm fractures in children. J Pediatr Orthop.

[24] Flynn JM, Jones KJ, Garner MR, Goebel J (2010). Eleven years experience in the operative management of pediatric forearm fractures. J Pediatr Orthop.

[25] Helenius I, Lamberg TS, Kääriäinen S, Impinen A, Pakarinen MP (2009). Operative treatment of fractures in children is increasing. A population-based study from Finland. J Bone Joint Surg Am.

[26] Cruz AI Jr, Kleiner JE, DeFroda SF, Gil JA, Daniels AH, Eberson CP (2017). Increasing rates of surgical treatment for paediatric diaphyseal forearm fractures: a national database Study from 2000 to 2012. J Child Orthop.

[27] Malheiros DS, Bárbara GH, Mafalda LG, Madureira JL Jr, Braga GF, Terra DL (2011). Floating elbow in children: a descriptive study of 31 cases attended in a reference center for pediatric trauma. Rev Bras Ortop.

[28] Karslı B, İnce K, Gönder N, Bozgeyik B, Kılınçoğlu V (2021). Surgery or conservative treatment of forearm in patients diagnosed with pediatric floating elbow? Retrospective analysis of 60 consecutive cases. Indian J Orthop.

[29] Heidari N, Wong J, Shetty S, Malaga-Shaw O, Barry M (20081). An unusual pattern of segmental forearm fracture in the immature forearm. Injury.

[30] Garg S, Dobbs MB, Schoenecker PL, Luhmann SJ, Gordon JE (2009). Surgical treatment of traumatic pediatric humeral diaphyseal fractures with titanium elastic nails. J Child Orthop.

[31] Kelly DM (2016). Flexible intramedullary nailing of pediatric humeral fractures: indications, techniques, and tips. J Pediatr Orthop.

[32] Lacher M, Schaeffer K, Boehm R, Dietz HG (2011). The treatment of supracondylar humeral fractures with elastic stable intramedullary nailing (ESIN) in children. J Pediatr Orthop.

